# Daratumumab-based immunotherapy vs. lenalidomide, bortezomib and dexamethasone in transplant-ineligible newly diagnosed multiple myeloma: a systemic review

**DOI:** 10.3389/fonc.2024.1286029

**Published:** 2024-01-25

**Authors:** Wenjiao Tang, Li Zhang, Yuhuan Zheng, Ling Pan, Ting Niu

**Affiliations:** Department of Hematology, Institute of Hematology, West China Hospital, Sichuan University, Chengdu, China

**Keywords:** myeloma, transplant-ineligible, immunotherapy, daratumumab, systematic review

## Abstract

**Background:**

Since no randomized controlled trials have directly compared the efficacy and safety of immunotherapy with daratumumab versus lenalidomide/bortezomib/dexamethasone (RVD) in the frontline treatment of transplant-ineligible newly diagnosed multiple myeloma (TIE-NDMM), this study systematically reviewed the clinical studies regarding immunotherapy with daratumumab and RVD regimen in the treatment of TIE-NDMM to explore the optimization direction of the best first-line therapy.

**Methods:**

The Cochrane Library, PubMed, Embase, and Web of Science databases were searched to collect studies on regimens containing daratumumab or RVD/RVD-lite for TIE-NDMM. Pooled and meta-analysis was then performed to compare the overall response rate (ORR), stringent complete remission (sCR) and CR rate, progression-free survival (PFS), overall survival (OS) and treatment-related discontinuation rate between daratumumab-containing immunotherapy regimen and RVD/RVD-lite regimen by using R 4.3.1 software.

**Results:**

Nine prospective clinical trials were included, including 1795 TIE-NDMM or NDMM without intent for immediate ASCT. Among them, 938 patients were treated with daratumumab-based immunotherapy and 857 with RVD/RVD-lite regimens. Meta-analysis results showed that The daratumumab-based regimen showed a significantly higher CR/sCR rate than RVD/RVD-lite for TIE-NDMM (47% vs. 24%, P<0.01). The median PFS of the daratumumab-based and RVD/RVD-lite groups were 52.6 months and 35.1 months respectively (HR 0.77, 95%CI, 0.66-0.90). The median OS of both groups was not reached, and there were no significant differences in OS between the two groups (HR 1.03, 95%CI, 0.86-1.23). The therapy discontinuation rate led by adverse events was significantly higher in the RVD/RVD-lite group than in the daratumumab-based regimen group for the TIE-NDMM (16% vs. 7%, P=0.03).

**Conclusion:**

This meta-analysis suggests that daratumumab-containing immunotherapy is superior to RVD in the depth of treatment efficacy, progression-free survival, and lower treatment-related discontinuation rates. Limited by the lack of head-to-head clinical trials, this conclusion needs to be verified by concurrent cohort studies.

## 1 Introduction

Multiple myeloma (MM) is a malignant clonal plasma cell disease ranking as the second most common hematological malignancy (1). The depth and duration of remission of first-line treatment are closely related to progression-free survival (PFS) and overall survival (OS), so it is critical to achieve the best outcome through the first-line treatment (2, 3). Effective induction therapy combined with autologous hematopoietic stem cell transplantation (ASCT) is still considered the standard first-line treatment for young patients with newly diagnosed multiple myeloma (NDMM), which can improve the response rate and significantly improve the survival of patients with MM (4). However, there is still a lack of consensus on a unified optimal and effective treatment for transplant-ineligible older MM patients with poor physical conditions and multiple complications (5). Meanwhile, the median age at diagnosis of MM is about 70 years old (6). With the aging of the population, the proportion of elderly MM patients will gradually increase, and the number of transplant-ineligible MM patients will increase progressively.

It has been proved that proteasome inhibitors and immune modulators combination therapy can achieve substantial effects in transplant-ineligible MM (TIEMM). For example, lenalidomide/bortezomib/dexamethasone (RVD) can improve the survival of TIEMM significantly compared to lenalidomide/dexamethasone (Rd) (7, 8). Besides, immunotherapy also made significant progress in MM (9), and research revealed that combination regimens such as daratumumab/lenalidomide/dexamethasone (DRd) and daratumumab/bortezomib/melphalan/dexamethasone (DVMP) achieved substantial efficacy in patients with TIEMM (10). Multiple guidelines recommend DRd, DVMP, and RVD as first-line treatment options for TIE-NDMM (4, 11). However, no randomized controlled trials have directly compared the efficacy and safety of immunotherapy with daratumumab-containing regimens versus RVD in the frontline treatment of TIE-NDMM. This study systematically reviewed the clinical studies of immunotherapy with daratumumab and RVD regimen for TIEMM to explore the optimization direction of the best first-line therapy for patients with TIEMM.

## 2 Materials and methods

### 2.1 Literature search strategy

Literatures were searched in PubMed, the Cochrane Library, Embase, and Web of Science databases to collect prospective clinical trials containing daratumumab-based regimens or RVD/RVD-lite regimens as the frontline therapy for TIE-NDMM. The timeframe for the searches was from the time of library construction until August 14, 2023. The investigation used subject terms and free words such as “myeloma”, “daratumumab”, “lenalidomide”, “bortezomib” and “transplant”. The detailed search strategies for each database were presented in the **Supplementary Files**.

### 2.2 Eligibility criteria

The inclusion criteria were as follows: 1) prospective studies; 2) the study population was TIE-NDMM of any age, gender, nationality, ethnicity and clinical stage; 3) the study treatment regimen includes a combination regimen based on daratumumab or RVD/RVD-lite (RVD reduction regimen) (12); 4) English literature and full texts reporting the endpoints including overall response rate (ORR), stringent complete remission (sCR) and CR rate, PFS, OS and treatment-related discontinuation rate. The exclusion criteria include that the article type was literature reviews, case reports or conference abstract, the article reported incomplete results, and the research subjects or protocols did not meet the requirements.

### 2.3 Data extraction

The literature underwent a rigorous screening process, with two independent evaluators extracting and cross-checking information. In instances of disagreement, a third party was consulted for additional judgment. To address any gaps in information, authors were contacted for supplementation whenever possible. The screening process involved an initial review of titles and abstracts, followed by a thorough examination of the full text. The final inclusion criteria were applied, eliminating obviously irrelevant literature. Data extraction mainly included: 1) essential characteristics of the study subjects; 2) specific details of the interventions and follow-up time, etc.; 3) critical elements of evaluating the risk of bias; 4) outcome indexes and outcome measurements of interest.

### 2.4 Assessment of quality

The evaluation of the quality of included randomized clinical trials hinged on the application of the Cochrane Collaboration Risk of Bias tool, a widely recognized method for assessing the risk of bias. In the case of non-randomized clinical trials, the Methodological Index for Non-Randomized Studies (MINORS) was utilized to measure the study quality.

### 2.5 Statistical analysis

The statistical analysis was executed using R 4.3.1 software. The relative risk (RR) was employed as the effect indicator accompanied by point estimates and 95% confidence intervals (CIs) for each effect size. Heterogeneity within the results of the included studies was evaluated using the χ2 test (α=0.1), and the magnitude of the heterogeneity was also quantitatively determined with *I* ^2^. In the absence of statistical heterogeneity among the study results, a fixed-effects model was applied for meta-analysis. Conversely, in the presence of statistical heterogeneity, an analysis of the source of heterogeneity was conducted. Subsequently, meta-analysis was performed using a random-effects model, with the exclusion of the influence of apparent clinical heterogeneity.

Survival data were analyzed using Engauge Digitizer to extract survival rates corresponding to the evaluation time points from the survival curves provided in the original article. The number of people at risk of events corresponding to each time period was extracted from the original literature or calculated using the methodology of Thiery (13) if not provided in the original article, assuming that the closure rate was constant over the follow-up period. The extracted survival data were combined, and combined survival curve estimates were generated using the metaSurvival program package (14).

## 3 Results

### 3.1 Literature search

**Figure 1:**
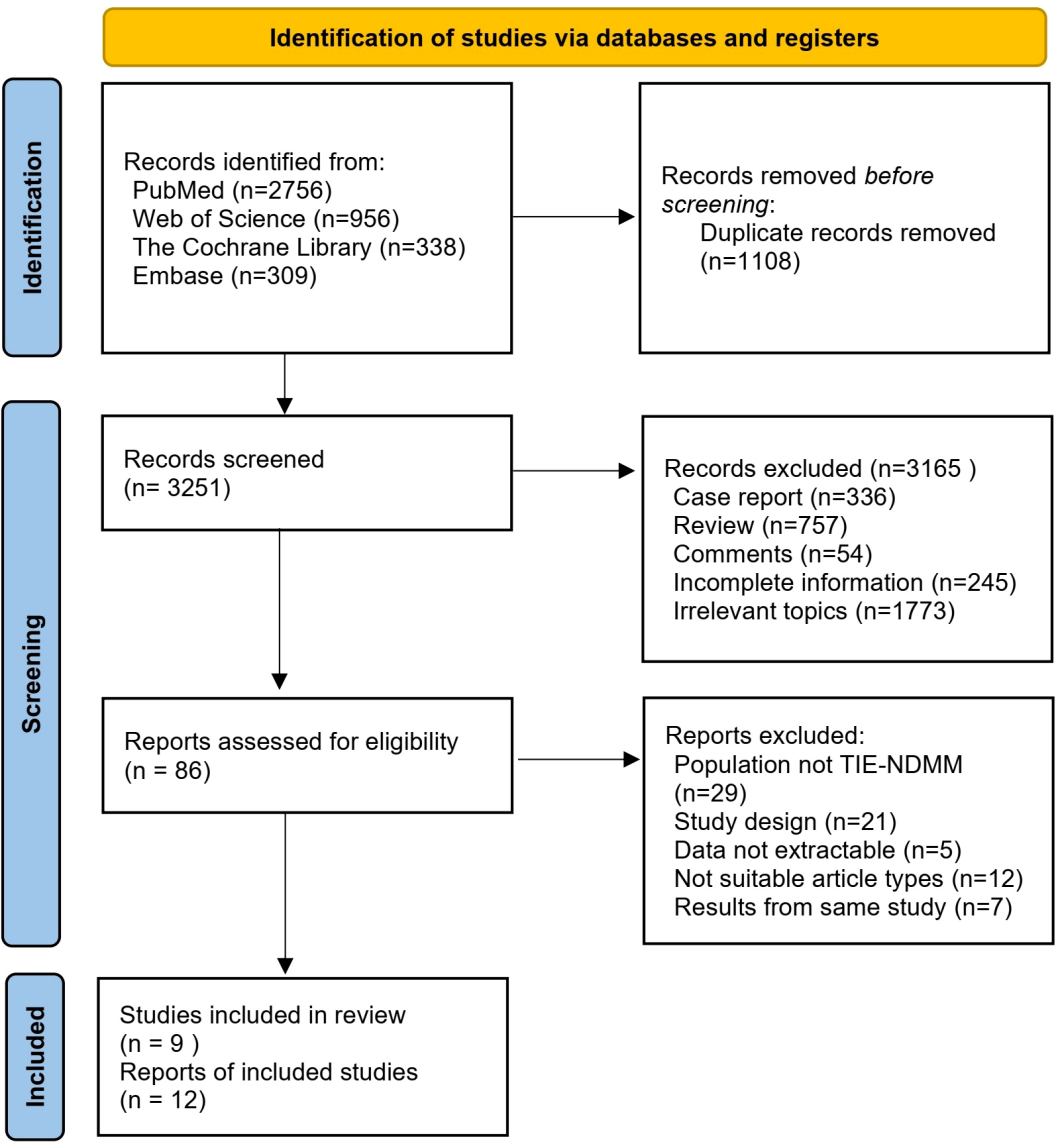
The detailed literature screening process and results.

A total of 4359 articles of related literature were initially retrieved. After a step-by-step screening process, nine prospective studies were finally included (8, 12, 15–21), including five randomized clinical trials and four single-arm studies. The detailed literature screening process and results are shown in **Figure 1**.

### 3.2 Study characteristics and quality assessment

The baseline characteristics of the nine studies, including five for the daratumumab-containing regimen group and four for the RVD/RVD-lite group, were summarized in **Table 1**. A total of 1795 TIE-NDMM or MM without intent for immediate ASCT were included, of which 938 patients were treated with daratumumab-containing immunotherapy regimens and 857 patients were treated with RVD/RVD-lite regimens. The daratumumab-containing regimen group comprised three DVMP cohorts and two DRd cohorts. The RVD/RVD-lite group formed two cohorts of RVD and two cohorts of RVD-lite.

**Table 1:** The baseline characteristics of the enrolled studies.

First author, year	ID	Trial name	Phase	Interventions	Sample size	Median age (range)	Renal insufficiency*	ECOG≥2 (N,%)	ISS Stage III (N, %)	High risk (N, %)	Median follow-up time (months)
Daratumumab-based regimen											
Facon 2021 (16)	NCT02252172	MAIA	3	DRd	368	73 (70~78)	162, 44.0%	63, 17%	107, 29%	48/319, 15%	56.2
Mateos 2020 (19)	NCT02195479	ALCYONE	3	DVMP	350	71 (40–93)	150, 42.9%	90, 25.7%	142, 40.6%	53/314, 19.9%	40.1
Fu 2023 (15)	NCT03217812	OCTANS	3	DVMP	146	69 (58–81)	63, 43.2%	25, 17.1%	41, 28.1%	28/145, 19.3%	12.3
Chari 2021 (17)	NCT03412565	PLEIADES	2	DVMP	67	75 (66–86)	NA	4, 6%	15, 22.4%	8/41, 19.5%	14.3
Takamatsu 2020 (18)	NCT02918331	MMY1006	1b	DRd	7	70 (66–81)	NA	0, 0%	0, 0%	2, 28.6%	11
RVD/RVD-lite											
Durie 2020 (8)	NCT00644228	SWOG S0777	3	RVD	235	63 (56-70)	11, 5%	24, 11%	77, 33%	NA	84
Kumar 2020 (21)	NCT01863550	ENDURANCE	3	RVD	542	64 (57–71)	6, 1.1%	60, 11%	138, 26%	NA	24
O’Donnell 2018 (12)	NCT01782963	NA	2	RVD lite	50	73 (65–91)	NA	7, 14%	18, 36%	6, 12%	30
Murakami 2022 (20)	UMIN000022008 jRCTs041180048	NA	2	RVD lite	30	72 (67–80)	15, 50%	5, 16.7%	10, 33.3%	NA	44.4

* Renal insufficiency was defined as baseline creatine clearance of no more than 60ml/min in the daratumumab-based regimen group and creatine concentration of more than 2mg/dL in the RVD/RVD-lite.

NA, not available.

**Figure 2:**
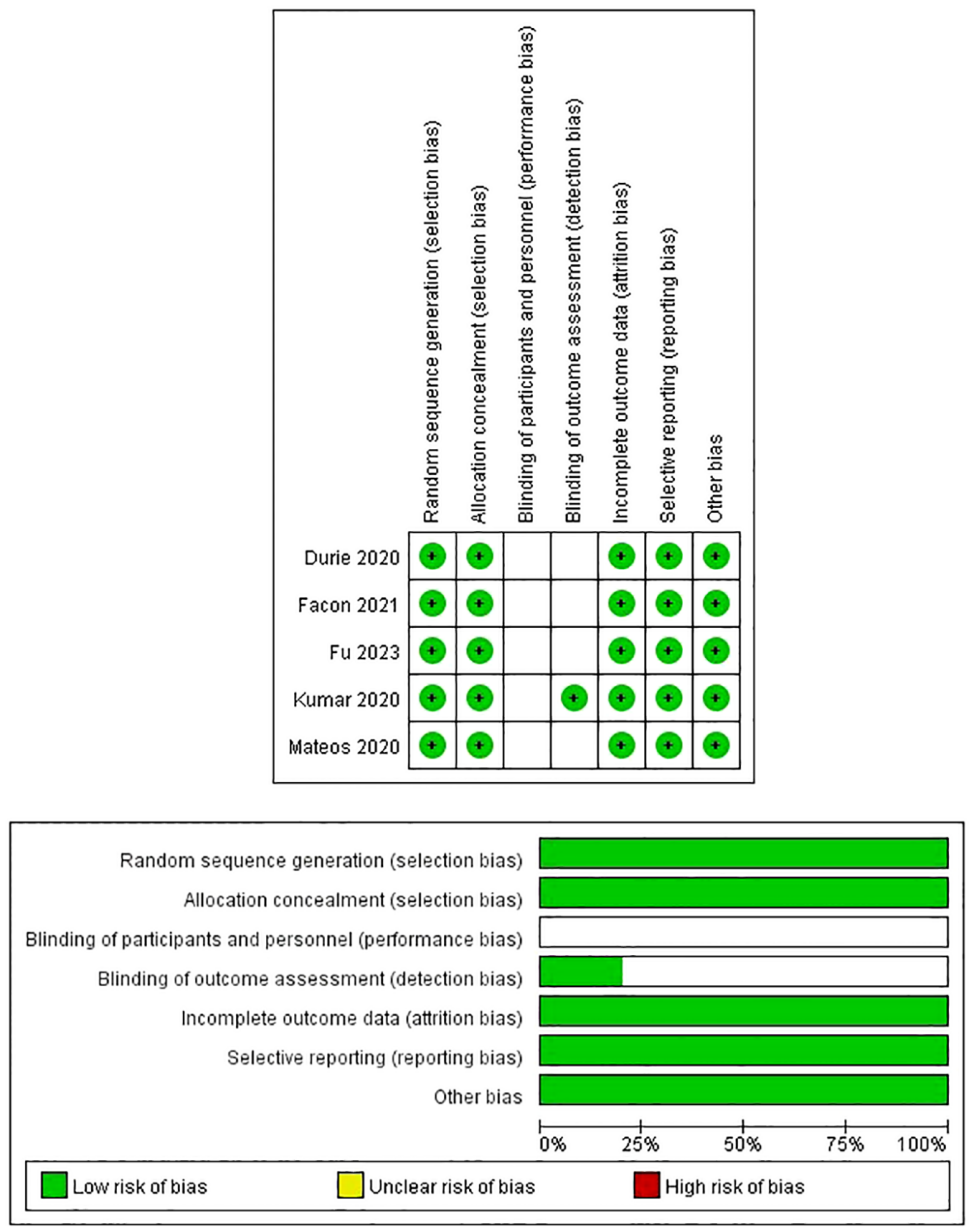
Risk of bias assessment in randomized clinical trials based on the Cochrane Collaboration Risk of Bias tool.

The detailed quality assessment of the included studies was presented in the **Supplementary Files**. The MINORS scores of four single arm studies ranged from 12 to 13 (**Table 2**). The quality assessment results of randomized clinical trials were satisfactory according to the Cochrane Collaboration’s tool (**Figure 2**).

**Table 2:** MINORS Index for non-randomized clinical trials.

Study	Q1	Q2	Q3	Q4	Q5	Q6	Q7	Q8	Total
Chari 2021 (17)	2	2	2	2	1	1	2	0	12
Takamatsu 2020 (18)	2	2	2	2	1	1	2	0	12
O’Donnell 2018 (12)	2	2	2	2	1	2	2	0	13
Murakami 2022 (20)	2	2	2	2	1	2	2	0	13

### 3.3 Pooled analysis and meta-analysis

#### 3.3.1 Efficacy

**Figure 3:**
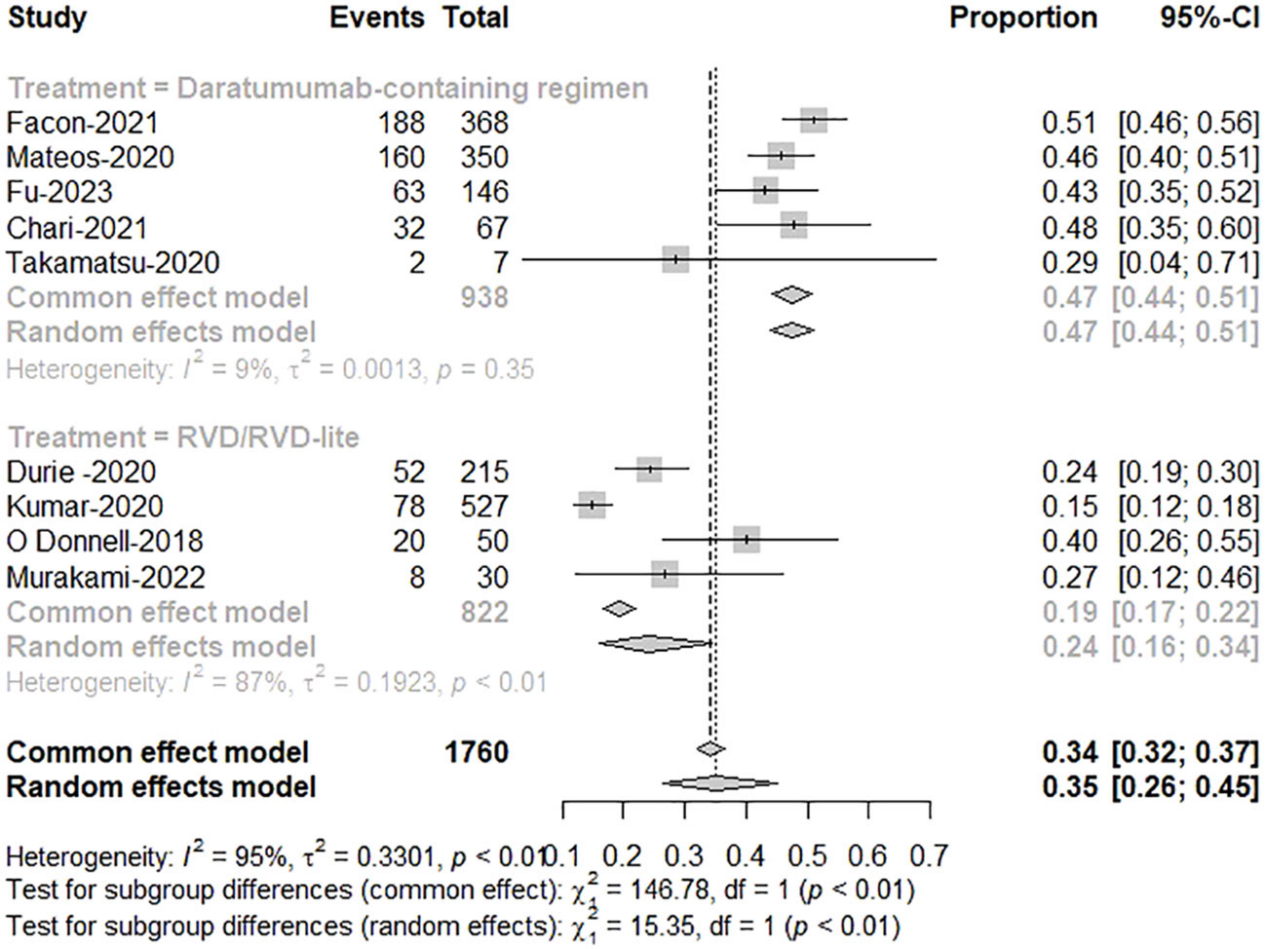
The meta-analysis of complete remission and better rates by subgroups of daratumumab-containing regimen and RVD/RVD-lite in transplant-ineligible newly diagnosed multiple myeloma.

**Figure 4:**
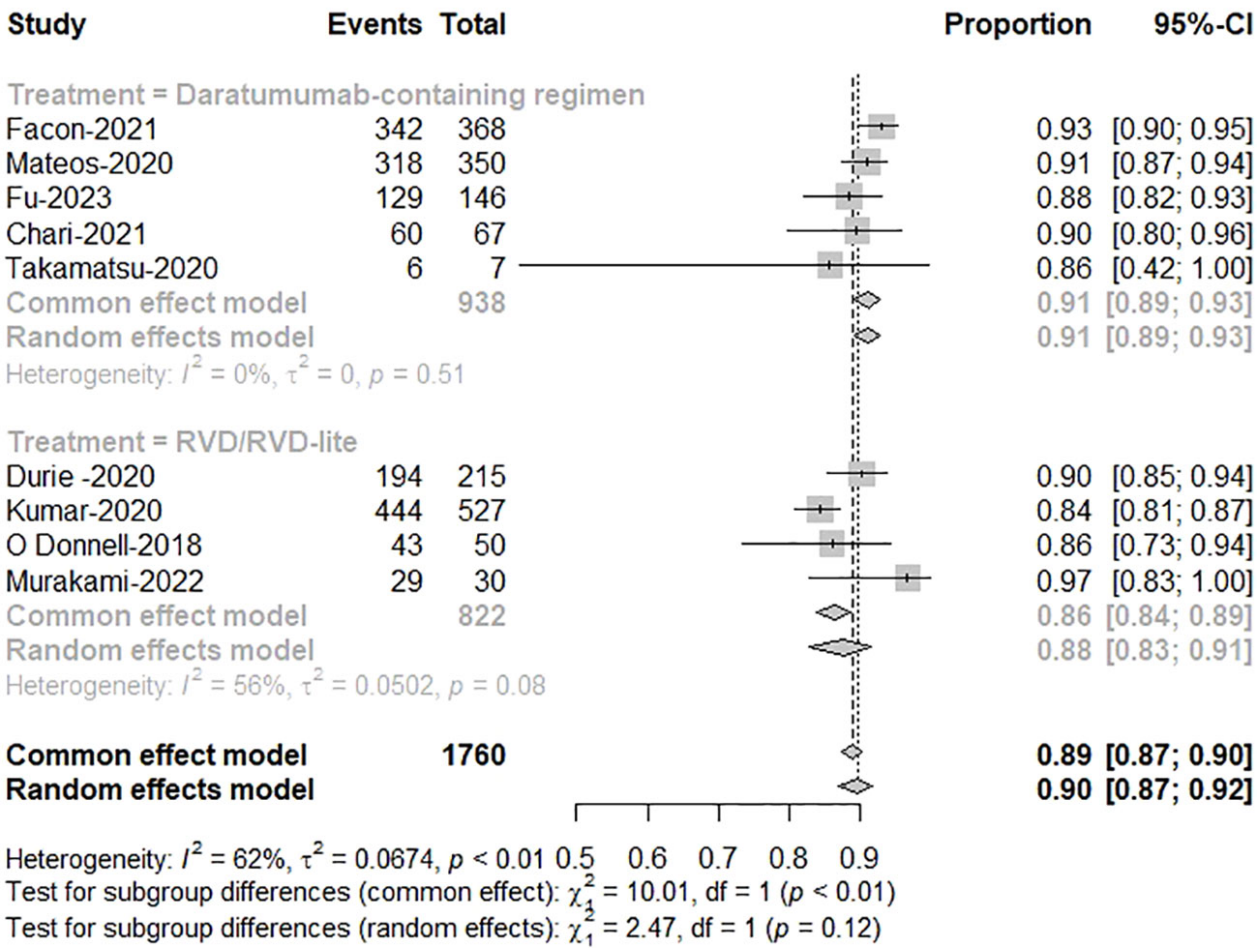
The meta-analysis of overall response rates by subgroups of daratumumab-containing regimen and RVD/RVD-lite in transplant-ineligible newly diagnosed multiple myeloma.

Five studies reported treatment responses for daratumumab-containing immunotherapy regimens and four for RVD/RVD-lite. The pooled analysis showed that the ORR was approximately 91% (95%CI, 89-93%) in the daratumumab-based regimen group and 88% (95%CI, 83-91%) in the RVD/RVD-lite group (**Figure 3**). The CR/sCR rate was approximately 47% (95%CI, 44-51%) in the daratumumab-based regimen group and 24% (95%CI, 16-34%) in the RVD/RVD-lite group. The daratumumab-based regimen showed a significantly higher CR/sCR rate than RVD/RVD-lite for TIE-NDMM (P<0.01) (**Figure 4**).

#### 3.3.2 Survival

**Figure 5:**
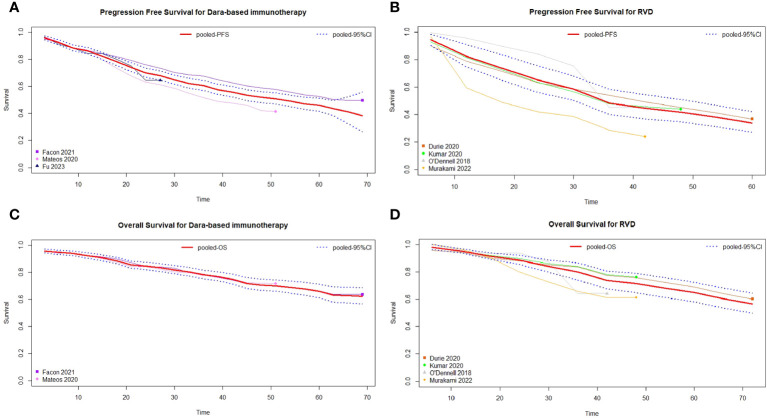
Pooled survival curves of transplant-ineligible newly diagnosed multiple myeloma. **(A)** Pooled progression free survival curves of daratumumab-containing regimen. **(B)** Pooled progression free survival curves of RVD/RVD-lite regimen. **(C)** Pooled overall survival curves of daratumumab-containing regimen. **(D)** Pooled overall survival curves of RVD/RVD-lite regimen.

Based on the studies with available PFS data, three studies were included in the daratumumab-based regimen group (15, 16, 19) and four in the RVD/RVD-lite group (8, 12, 20, 21). Survival curves for the PFS of the two groups are shown in **Figures 5A**, **B**. The median PFS of the daratumumab-based regimen group and RVD/RVD-lite group were 52.6 (95%CI, 44.0-59.5) months and 35.1 (95%CI, 29.4-47.1) months respectively (HR 0.77, 95%CI, 0.66-0.90). There was no significant difference in the one-year PFS rate (86% vs. 82%, P=0.43) and two-year PFS rate (68% vs. 65%, P=0.64).

For the meta-analysis of OS, two studies with available data were included in the daratumumab-based regimen group (16, 19) and four in the RVD/RVD-lite group (8, 12, 20, 21). The median OS of both groups was not reached, and there were no significant differences in OS between the two groups (HR 1.03, 95%CI, 0.86-1.23) (**Figures 5C, D**).

#### 3.3.3 Treatment-related discontinuation

**Figure 6:**
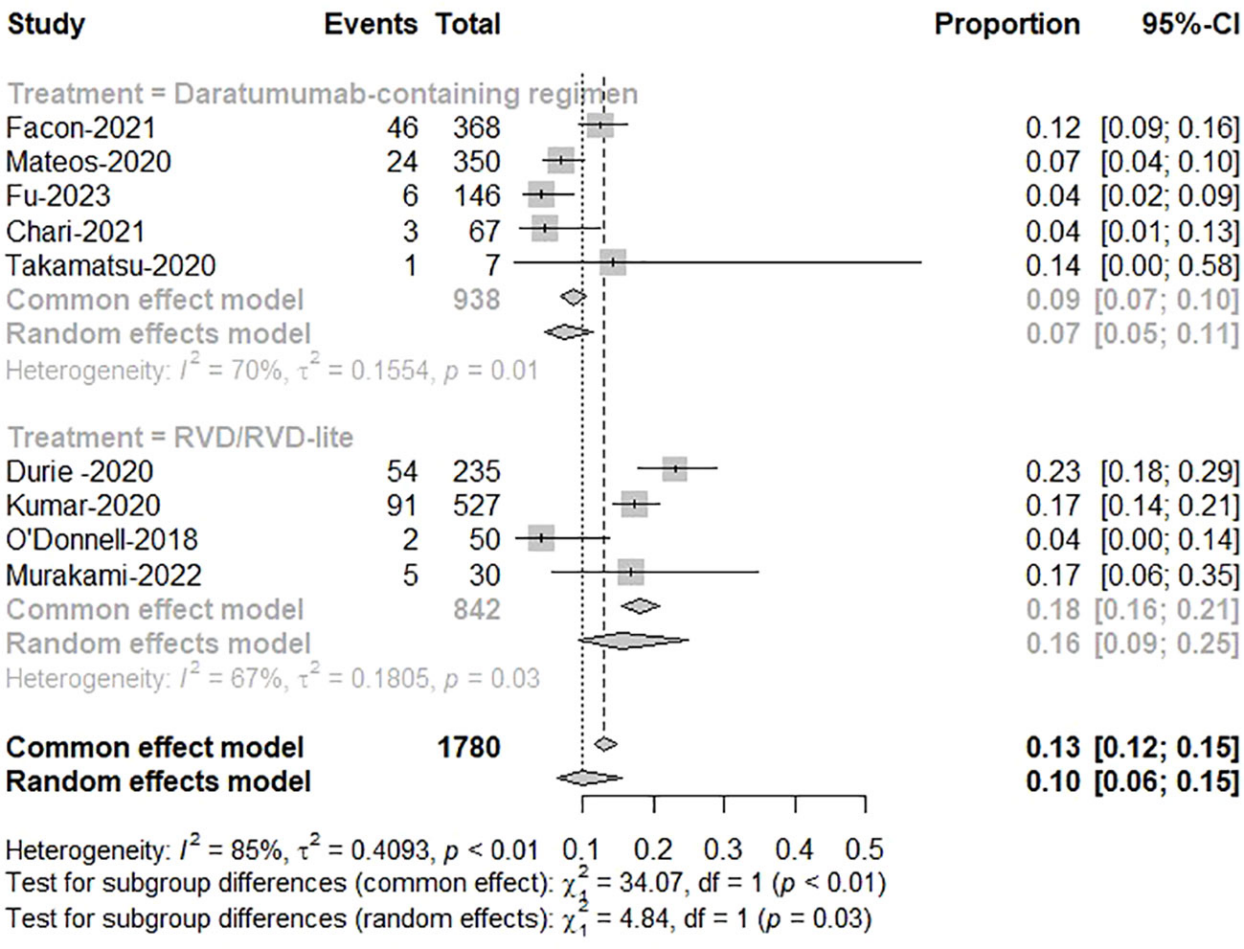
The meta-analysis of therapy discontinuation rate due to adverse events by subgroups of daratumumab-containing regimen and RVD/RVD-lite in transplant-ineligible newly diagnosed multiple myeloma.

The pooled analysis showed the therapy discontinuation rate leading by adverse events was significantly higher in the RVD/RVD-lite group than in the daratumumab-based regimen group for the TIE-NDMM (16% vs. 7%, P=0.03) (**Figure 6**).

## 4 Discussion

Elderly MM patients often have many comorbidities and generally cannot tolerate intensive chemotherapy or ASCT, resulting in a poorer prognosis than younger patients. The optimal treatment regimen for TIE-NDMM is currently unclear (22–24). The treatment choice must balance efficacy and toxicity to achieve the best possible effectiveness with the least toxic regimen as far as possible.

The clinical trial SWOG S0777 showed that RVD significantly improved PFS and OS in patients with TIE-NDMM compared to Rd (7). Still, in MM patients 65 years and older, the OS benefit was insignificant with the RVD regimen, and grade 3 and higher neurotoxicity and gastrointestinal adverse events were significantly increased (8). Therefore, there is still a need to explore safer and more effective therapeutic regimens for elderly patients unsuitable for transplantation. Both the ALCYONE and MAIA clinical studies investigated the efficacy and safety of the standard treatment regimen combined with daratumumab for TIE-NDMM, and the results demonstrated that the immunotherapy enhanced efficacy and prolonged survival (25). However, no randomized controlled trials directly compare the efficacy and safety of daratumumab-based immunotherapy regimens with RVD/RVD lite for TIE-NDMM. Due to the lack of head-to-head comparative studies and considering the closer screening conditions for clinical trial enrollment, only prospective clinical trial study data were included in this paper, and no real-world data were included for systematic evaluation and reanalysis. The pooled analysis results showed that for patients with TIE-NDMM, daratumumab-based immunotherapy was more advantageous in increasing the depth of therapeutic response and did not increase the treatment-related discontinuation rate. Meanwhile, the systematic evaluation showed that the daratumumab-based immunotherapy had a more pronounced benefit over the RVD/RVD lite regimen in improving PFS in patients with TIE-NDMM but no significant difference in improving OS.

The PEGASUS study indirectly compared DRd and RVD regimens for the treatment of TIE-NDMM based on the results of the MAIA clinical trial and data from the Flatiron Health database, which showed that DRd reduced the risk of disease progression and death by 32% compared with the RVD regimen (26). In addition, a network meta-analysis comparing different frontline therapies for TIE-NDMM showed that DRd and DVMP were more advantageous in improving PFS. Still, the results of DVMP and RVD were similar regarding the benefits in enhancing OS (27). These findings and our study support that compared to RVD regimens, daratumumab-based immunotherapy is more beneficial in improving PFS but similar in prolonging OS for TIE-NDMM. The OS may be affected by salvage therapies.

Regarding exploring the timing of combination daratumumab treatment, Fonseca et al (28) estimated patient survival outcomes by modeling three clinical treatment modalities based on treatment guideline recommendations for TIE-NDMM. They found that the DRd sequential pomalidomide/carfilzomib regimen as second-line therapy had the highest estimated 5-, 10-, and 15-year OS rates, and daratumumab-based regimen as the frontline therapy for TIE-NDMM had a more pronounced OS benefit than as second-line treatment (28). However, the study used combination attrition rates to estimate outcomes, which were variable factors in different studies (29–32). Further clinical studies are needed to validate the timing of daratumumab use.

On the other hand, there is still uncertainty about the subgroups of TIE-NDMM benefitting the most from the daratumumab-based therapy, such as the frail elderly, renal insufficiency, cardiac amyloidosis, high-risk groups, etc. More clinical data are needed to help decision-making about the risks and benefits of adopting immunotherapy. The meta-analysis by Costa et al. found that the combined daratumumab with standard treatment prolonged the PFS in the cytogenetically high-risk group compared to the control regimen for both NDMM and relapsed-refractory MM (33). The combined analysis of the MAIA and ALCYONE studies found that the cytogenetically high-risk subgroup TIE-NDMM had a CR rate of 41.6% with the daratumumab-containing regimen, with a median PFS of 21.2 months and a 41% reduction in the risk of disease progression or death compared to the control regimen (34). However, none of the above studies analyzed a direct head-to-head comparison of daratumumab-based therapy with the RVD regimen in the high-risk TIE-NDMM.

Still, there exist limitations in this systematic analysis. Due to the lack of head-to-head comparison of randomized controlled trials of daratumumab-containing regimens and RVD regimens for the treatment of TIE-NDMM, the conclusions of this systematic evaluation are based on single-arm analysis and need to be verified by contemporaneous cohort studies. Secondly, some of the included studies did not use the correct blinding method, and the outcome judgments or measurements will be affected, which may result in bias. Due to the limitations of the original data, it is impossible to carry out the comparative subgroup analyses of the two regimens. Meanwhile, endpoints such as time to next treatment and PFS2 are also critical to assess the treatment benefits. However, the trials did not report these results, and it still needs a longer follow-up time to test this.

In summary, daratumumab-containing regimens have advantages over RVD regimens for TIE-NDMM in terms of therapeutic efficacy, prolongation of PFS, and reduction of treatment-related discontinuation rates. With the progress of new drugs, a series of clinical trials on daratumumab-based immunotherapy and RVD/RVD-lite regimens for treating TIE-NDMM are still underway.

## Data availability statement

The raw data supporting the conclusions of this article will be made available by the authors, without undue reservation.

## Author contributions

WT: Data curation, Funding acquisition, Investigation, Writing – original draft. LZ: Conceptualization, Writing – review & editing. YZ: Data curation, Writing – review & editing. LP: Investigation, Writing – review & editing. TN: Supervision, Writing – review & editing.
