# Supplementary material for: Daratumumab-based immunotherapy vs. lenalidomide, bortezomib and dexamethasone in transplant-ineligible newly diagnosed multiple myeloma: a systemic review
Source: Front Oncol. 2024 Jan 25;14:1286029. doi: 10.3389/fonc.2024.1286029 (PMC10850248; doi:10.3389/fonc.2024.1286029)
Supplement: Supplementary file 1 [file DataSheet_1.docx]

Supplementary Material

**Detailed search strategies in PubMed, the** **Cochrane Library, Embase, and Web of Science databases**

1. Pubmed

#1 "multiple myeloma"[MeSH Terms] OR ("multiple"[All Fields] AND "myeloma"[All Fields]) OR "multiple myeloma"[All Fields] OR "myeloma"[All Fields] OR "myelomas"[All Fields] OR "myeloma s"[All Fields]

#2 "lenalidomid"[All Fields] OR "lenalidomide"[Supplementary Concept] OR "lenalidomide"[All Fields] OR "lenalidomide"[MeSH Terms] OR "lenalidomide s"[All Fields]

#3 "bortezomib"[Supplementary Concept] OR "bortezomib"[All Fields] OR "bortezomib"[MeSH Terms] OR "bortezomib s"[All Fields]

#4 "daratumumab"[Supplementary Concept] OR "daratumumab"[All Fields]

#5 "transplantability"[All Fields] OR "transplantable"[All Fields] OR "transplantated"[All Fields] OR "transplantating"[All Fields] OR "transplantation"[MeSH Terms] OR "transplantation"[All Fields] OR "transplantations"[All Fields] OR "transplanted"[All Fields] OR "transplanting"[All Fields] OR "transplantation"[MeSH Subheading] OR "transplantation s"[All Fields] OR "transplanter"[All Fields] OR "transplanters"[All Fields] OR "transplantion"[All Fields] OR "transplants"[MeSH Terms] OR "transplants"[All Fields] OR "transplant"[All Fields]

#6 #2 OR #3 OR #4

#7 #1 AND #5 AND #6

2. Web of science

#1 ((((TS=(daratumumab)) OR TS=(lenalidomide)) OR TS=(bortezomib))) NOT (SILOID==("PPRN"))

#2 (TS=(myeloma)) NOT (SILOID==("PPRN"))

#3 (TS=(transplant)) NOT (SILOID==("PPRN"))

#4 (#3 AND #2 AND #4) AND ((DT==("ARTICLE")) NOT (SILOID==("PPRN") OR DT==("REVIEW") OR DT==("CASE REPORT" OR "BOOK" OR "CORRECTION" OR "REFERENCE MATERIAL" OR "NEWS")))

3. The Cochrane Library trial

#1 ("daratumumab" or "lenalidomide" or "bortezomib").mp. [mp=title, original title, abstract, floating sub-heading word, mesh headings, heading words, keyword]

#2 multiple myeloma/ or leukemia, plasma cell/

#3 transplant.mp.

#4 #1 AND #2 AND #3

4. Embase

#1 multiple myeloma/ or myeloma/

#2 daratumumab/ct [Clinical Trial]

#3 lenalidomide/ct [Clinical Trial]

#4 bortezomib/ct [Clinical Trial]

#5 #2 OR #3 OR #4

#6 #1 AND 5#

#7 transplant.mp. or transplantation/

#8 #6 AND 7#
